# An introduction to the mathematical structure of the Wright–Fisher model of population genetics

**DOI:** 10.1007/s12064-012-0170-3

**Published:** 2012-12-14

**Authors:** Tat Dat Tran, Julian Hofrichter, Jürgen Jost

**Affiliations:** 1 Max Planck Institute for Mathematics in the Sciences, 04103 Leipzig, Germany; 2 Department of Mathematics, Leipzig University, 04081 Leipzig, Germany; 3 Santa Fe Institute for the Sciences of Complexity, Santa Fe, NM 87501 USA

**Keywords:** Random genetic drift, Wright–Fisher model, Fokker–Planck equation

## Abstract

In this paper, we develop the mathematical structure of the Wright–Fisher model for evolution of the relative frequencies of two alleles at a diploid locus under random genetic drift in a population of fixed size in its simplest form, that is, without mutation or selection. We establish a new concept of a global solution for the diffusion approximation (Fokker–Planck equation), prove its existence and uniqueness and then show how one can easily derive all the essential properties of this random genetic drift process from our solution. Thus, our solution turns out to be superior to the local solution constructed by Kimura.

## Introduction

In population genetics, one considers the effects of recombination, selection, mutation, and perhaps others like migration on the distribution of alleles in a population, see e.g. (Ewens 2004; Bürger 2000; Rice 2004) as mathematical textbook references. The most basic and at the same time important model is the Wright–Fisher model for random genetic drift [developed implicitly by Fisher (1922) and explicitly by Wright (1931)]. In its simplest version—the one to be treated in the present paper—it is concerned with the evolution of the relative frequencies of two alleles at a single diploid locus in a finite population of fixed size with non-overlapping generations under the sole force of random genetic drift, without any other influences like mutations or selection. The model can be generalised—and so can our approach—to multiple alleles, several loci, with mutations, selections, spatial population structures, etc, see the above references. To find an exact solution (for the approximating diffusion process for the probability densities of the allele frequencies described by a Fokker–Planck equation) from which the properties of the resulting stochastic process can be deduced, however, is difficult. For the basic two-allele case, this was first achieved in the important work of Kimura (1955), and he then went on to treat the case of several alleles (Kimura 1955, 1956). His solution, however, is local in the sense that it does not naturally incorporate the transitions resulting from the irreversible loss of one or several of the alleles initially present in the population. Consequently, the resulting probability distribution does not integrate to 1, and it is difficult to read off the quantitative properties of the process from his solution.

In the present paper, we introduce and describe a new global approach. This approach is mathematically more transparent than Kimura’s scheme. We prove the existence of a unique such global solution (see Theorem 3.7), and we can deduce all desired quantities of the underlying stochastic process from our solution. The purpose of the present paper thus is to display the method in the simplest case, that of two alleles at a single locus, so that the structure becomes clear. The case of multiple alleles is presented in our companion paper (Tran et al. 2000) on the basis of the first author’s thesis, and further generalisations will be systematically developed elsewhere within the mathematical framework of information geometry (Amari and Nagaoka 2000) and more specifically (Ay and Jost 2000; Jost 2000) on the basis of the second author’s thesis.

## The Wright–Fisher model

We consider a diploid population of size *N*. At a given locus, there could be either one of the two alleles *A*_1_,*A*_2_. Thus, an individual can be a homozygote of type *A*_1_ *A*_1_ or *A*_2_ *A*_2_ or a heterozygote of type *A*_1_ *A*_2_ or *A*_2_ *A*_1_—but we consider the latter two as the same—at the locus in question. The population reproduces in discrete time steps, and each individual in generation *n* + 1 inherits one allele from each of its parents. When a parent is a heterozygote, each allele is chosen with probability 1/2. Here, for each individual in generation *t* + 1, randomly two parents in generation *n* are chosen. Thus, the alleles in generation *n* + 1 are chosen by random sampling with replacement from the ones in generation *n*. The quantity of interest is the number *Y*_*n*_ of alleles *A*_1_ in the population at time *n*. This number then varies between 0 and 2*N*. The transition probability then is

1 $$ {\mathbb{P}}(Y_{n+1}=j|Y_n=i)={2N \choose j} \left(\frac{i}{2N}\right)^j\left(1- \frac{i}{2N}\right)^{2N-j}\quad\hbox {for } i,j=0,\dots,2N. $$

whenever *Y*_*n*_ takes the value 0 or 2*N*, that is, if either the allele *A*_1_ or *A*_2_ will disappear, it will stay there for all future times. Eventually, this will happen almost surely.

This is the basic model. One can then derive expressions for the expected time for the allele *A*_1_ to become either fixed, that is, *Y*_*n*_ = 2*N*, or become extinct, *Y*_*n*_ = 0, given its initial number *Y*_0_.

An important idea, first applied in Wright (1945), then is to rescale time and population size via

2 $$ t=\frac{n}{2N},\quad X_t=\frac{Y_t}{2N}, $$

and then consider the limit $N\to \infty.$ The rescaling of (2) yields a discrete Markov chain *X*_*t*_ valued in $\{0, \frac{1}{2N},\ldots,1\}$ with *t* = 1 now corresponding to 2*N* generations. One readily verifies that the expectation values for the variation across generations satisfy

3 $$ \begin{aligned} X_0&=p=\frac{i_0}{2N};\\ {\mathbb{E}}(\delta X_t)&=0;\\ {\mathbb{E}}(\delta X_t)^2&=X_t(1-X_t) \delta t;\\ {\mathbb{E}}(\delta X_t)^k&= o(\delta t)\;\hbox {for }k\ge 3. \end{aligned} $$

A basic idea of our approach is to consider the *k*th moment *m*_*k*_(*t*) of the distribution about zero at the (2*Nt*)th generation, i.e.

4 $$ m_k(t)={\mathbb{E}}(X_t)^{k} $$

We have

5 $$ m_k(t+1)={\mathbb{E}}(X_t+\delta X_t)^{k} $$

Expanding the right hand side and noting (3) we obtain the following recursion formula

6 $$ m_k(t+1)=\left\{1-\frac{k(k-1)}{2}\right\} m_k(t)+\frac{k(k-1)}{2}m_{k-1}(t) $$

when we assume that the population number *N* is so large that we can neglect all terms of order at least $\frac{1}{N^2}.$ Under this assumption, the moments change very slowly per generation and we can replace the above system (6) by the system of differential equations

7 $$ \dot{m}_k(t)=-\frac{k(k-1)}{2} m_k(t)+\frac{k(k-1)}{2}m_{k-1}(t), $$

where the dot denotes a derivative w.r.t. the variable *t*.

These formulae now guide us in finding a continuous process that well approximates the above discrete process. We seek a continuous Markov process {*X*_*t*_}_*t* ≥ 0_ valued in [0,1] with the same conditions as (3) and (7). The conditions (3) imply (see for example Ewens 2004, p. 137, for a derivation) that the probability density function *u*(*x*, *t*) of this continuous process is a solution of the Fokker–Planck (Kolmogorov forward) equation

8 $$ \left\{\begin{array}{l} u_t(x,t)=\frac{1}{2}\frac{\partial^2}{\partial x^2}\left(x(1-x)u(x,t)\right)\hbox { in }(0,1)\times (0,\infty),\\ u(x,0)=\delta_p(x) \hbox { in }(0,1) \end{array}\right. $$

where we now use the notation $u_t:=\frac{\partial}{\partial t}u(x,t)$ for the partial derivative w.r.t. the time variable *t*. The coefficient *x*(1 − *x*) in (8) comes from (3) and δ_*p*_ denotes the Dirac delta function at *p*. For the definition of this delta function, we use the product

$$ (f,g):=\int\limits_0^1 f(x)g(x)\,{\hbox{d}}x $$

for square integrable functions ${f,g:[0,1]\to \mathbb{R}}$ on the unit interval (this will be described in more detail in “Existence and uniqueness of solutions”), and we then put

$$ (\delta_p,\phi):= \phi(p) $$

whenever ${\phi:[0,1]\to \mathbb{R}}$ is a continuous function.^1^

Let us also explain the interpretation of (8) for those not sufficiently versed in this mathematical formalism. The initial condition *u*(*x*,0) = δ_*p*_(*x*) then simply says that at time 0, the relative frequency of allele *A*_1_ is precisely *p*, without any uncertainty (this assumption is not essential, however, and the scheme works also for more general initial condition involving uncertainty about the initial distribution of the alleles). Subsequently, this allele frequence evolves stochastically, according to the equation $u_t(x,t)=\frac{1}{2}\frac{\partial^2}{\partial x^2}\left(x(1-x)u(x,t)\right),$ and therefore, for *t* > 0, we no longer know the precise value of this relative frequency, but only its probability density given by *u*(*x*, *t*). That is, for every *x*, the probability density that the allele frequency at time *t* has the value *x* is given by *u*(*x*, *t*).

In the continuum limit, the *k*th moment becomes $\int_0^1 u(x,t)x^k\,{\hbox{d}}x,$ and the condition (7) then implies

$$ \begin{aligned} (u_t,x^k)&=\int\limits_0^1\frac{\partial}{\partial t} u(x,t)x^k\,{\hbox{d}}x\\ &=\frac{{\hbox{d}}}{{\hbox{d}}t}\int\limits_0^1 u(x,t)x^k\,{\hbox{d}}x\\ &=\left(u,-\frac{k(k-1)}{2} x^k+\frac{k(k-1)}{2} x^{k-1}\right)\\ &=\left(u,\frac{1}{2}x(1-x)\frac{\partial^2}{\partial x^2}(x^k)\right),\quad \forall k\ge 0. \end{aligned} $$

Since the polynomials are dense in the space of (square integrable) functions, this yields

9 $$ (u_t,\phi)=\left(u,\frac{1}{2}x(1-x)\frac{\partial^2}{\partial x^2} \phi\right) $$

for all square integrable functions ${\phi:[0,1]\to \mathbb{R}}$ that are twice differentiable in the open interval (0,1).

This leads to our concept of a solution of the Fokker–Planck equation in

### **Definition 2.1**

We call $u\in H$ a solution of the Fokker–Planck equation associated with the Wright–Fisher model if

10 $$ u_t=Lu \hbox { in }(0,1)\times (0,\infty), $$

11 $$ u(x,0)=\delta_p(x) \hbox { in }(0,1); $$

12 $$ (u_t,\phi)=(u,L^* \phi),\quad \forall \phi\in H_0, $$

for all square integrable functions ${\phi:[0,1]\to \mathbb{R}}$ that are twice differentiable in the open interval (0, 1), with the differential operator

13 $$ Lu(x):=\frac{1}{2}\frac{\partial^2}{\partial x^2}\left(x(1-x)u(x)\right) $$

and its formal adjoint

14 $$ L^*\phi(x)=\frac{1}{2}x(1-x)\frac{\partial^2}{\partial x^2}\phi(x). $$

This solution concept will allow us to prove the existence of a unique solution from which we can then derive all features of interest of the Wright–Fisher process. We should point out that (12) is not just the integration by parts of (10), but also includes the boundary behaviour (of course, this may not be overt, but the mathematical trick here is to represent this boundary behaviour in an implicit form best suited for formal manipulation). It, thus, reflects transitions from the presence of both alleles to the irreversible loss of one of them. This is the crucial difference to Kimura’s (1955) solution concept and the key for the properties of our solution.

## Existence and uniqueness of solutions

We shall now apply a familiar mathematical scheme for the construction of a solution of a differential equation, an expansion in terms of eigenfunctions of the differential operator involved. For our problem, as formalised in Definition 2.1, these eigenfunctions can be constructed from a classical family of polynomials, the Gegenbauer polynomials, which we shall now introduce.

### Preliminaries

For the sequel, we shall need some more notation. We need the function spaces

$$ H_0:= C^\infty[0,1], $$

$$ H:= \left\{ f:[0,1]\to [0,\infty] \; \hbox {measurable with } \int\limits_{[0,1]}f(x)g(x)\,{\hbox{d}}x <\infty,\quad \forall g\in H_0\right\}, $$

with the scalar product

$$ (f,g):= \int\limits_{[0,1]}f(x)g(x)\,{\hbox{d}}x,\quad \forall f\in H, g\in H_0. $$

To construct solutions in terms of expansions, we shall need a special case of the Gegenbauer polynomials [named after Leopold Gegenbauer (1849–1903)].^2^ The polynomials *Y*_*m*_(*z*) we need are defined in terms of their generating function

$$ \frac{1}{(1-2zt+t^2)^\frac{3}{2}}=\sum\limits_{m\ge 0} Y_m(z) t^m. $$

#### **Lemma 3.1**

(Suetin 2001)

- *The Gegenbauer polynomials satisfy the recurrence relation*
  $$ \begin{aligned} Y_0(z) &= 1\\ Y_1(z) &= 3 z\\ Y_n (z) &=\frac{1}{n}\left[2z(n+\frac{1}{2})Y_{n-1} (z) - (n+1)Y_{n-2} (z)\right]. \end{aligned} $$
- *The Gegenbauer polynomials solve the differential equation*
  15 $$ (1-z^{2})y^{\prime\prime}-4zy^{\prime}+n(n+3)y=0. $$

#### **Lemma 3.2**

[Abramowitz (1965), p. 774] *The polynomials* *Y*_*m*_ *are orthogonal polynomials on the interval* [−1,1] *with respect to the weight function* (1 − *z* ^2^):

16 $$ \int\limits_{-1}^1 (1-z^2)Y_m(z)Y_n(z)\,{\hbox{d}}z=0\quad\hbox{for }\ m\neq n. $$

### Auxiliaries

#### **Lemma 3.3**

*For all* *m* ≥ 0 *we have*

$$ LX_m=-\lambda_m X_m, \; in \; H_0 $$

*with*

$$ \lambda_m:= \frac{(m+1)(m+2)}{2}. $$

#### *Proof*

Putting *z* = 1 − 2*x* implies that

$$ Y_m(z):=X_m(x) $$

is a Gegenbauer polynomial and therefore solves (15),

$$ (1-z^2)\frac{\partial^2}{\partial z^2}Y_m(z)-4z\frac{\partial}{\partial z}Y_m(z)+m(m+3)Y_m(z)=0. $$

This is equivalent to

$$ \begin{aligned} x(1-x)\frac{\partial^2}{\partial x^2}X_m(x)-2(1-2x)\frac{\partial}{\partial x}X_m(x)+m(m+3)X_m(x)&=0,\\ \Longleftrightarrow \frac{\partial^2}{\partial x^2}( x(1-x)X_m(x))&=-(m+1)(m+2)X_m,\\ \Longleftrightarrow LX_m&=-\lambda_m X_m. \end{aligned} $$

This completes the proof.$\square$

In the sequel, we shall use the abbreviation

$$ w(x):=x(1-x). $$

#### **Lemma 3.4**

*If* *X* *is an eigenvector of* *L* *corresponding to the eigenvalue* λ *then* *wX* *is an eigenvector of* *L* ^*^ *corresponding to the eigenvalue* λ.

#### *Proof*

Assume that *X* is an eigenvector of *L* for the eigenvalue λ, i.e.

$$ \frac{1}{2}\frac{\partial^2}{\partial x^2}(wX)=-\lambda X. $$

Multiplying both sides by *w* yields

$$ L^*(wX)=\frac{1}{2}w\frac{\partial^2}{\partial x^2}(wX)=-\lambda (wX). $$

This completes the proof. $\square$

#### **Lemma 3.5**

*The spectrum of the operator* *L* *is*

$$ Spec(L)=\bigcup_{m\ge 0} \left\{\lambda_m=\frac{(m+1)(m+2)}{2}\right\} := \Uplambda, $$

*and the eigenvector of* *L* *corresponding to* λ_*m*_ *is the Gegenbauer polynomial* *X*_*m*_(*x*) *(up to a constant)*.

#### *Proof*

From Lemma 3.3 we have *L*(*X*_*m*_) =  − λ_*m*_ *X*_*m*_ in *H*_0_. So, $\Uplambda\subseteq Spec(L).$ Conversely, we shall prove that $\lambda \notin \Uplambda$ is not an eigenvalue of *L*. In fact, assume that there is some $X \in H_0$ with $L X= -\lambda X \in H_0.$ Because {*X*_*m*_}_*m* ≥ 0_ is a basis of *H*_0_, we can represent *X* by $X=\sum\nolimits_{m=0}^\infty d_m X_m.$ Then

$$ \sum\limits_{m=0}^\infty d_m (-\lambda_m X_m)=\sum\limits_{m=0}^\infty d_m L(X_m)=L\left(\sum\limits_{m=0}^\infty d_m X_m\right)=-\lambda \sum\limits_{m=0}^\infty d_m X_m. $$

For any *n* ≥ 0, we can multiply this relation by *wX*_*n*_ and then integrate on [0,1]. From the orthogonality (16) with respect to the weight function *w*, we obtain

$$ d_n \lambda_n (X_n,wX_n)=d_n \lambda (X_n,wX_n). $$

Because (*X*_*n*_,*wX*_*n*_)≠ 0 and λ≠ λ_*n*_, then *d*_*n*_ = 0, ∀ *n* ≥ 0. Therefore, *X* = 0, i.e. λ is not an eigenvalue of *L*. Thus

$$ {\hbox{Spec}}(L)=\Uplambda. $$

Similarly, if *X* is an eigenvector of *L* for the eigenvalue λ_*m*_, we will prove that *X* = *c* *X*_*m*_. In fact, representing $X=\sum\nolimits_{n=0}^\infty d_n X_n,$ it follows that

$$ \sum\limits_{n=0}^\infty d_n (-\lambda_n X_n)=\sum\limits_{n=0}^\infty d_n L(X_n)=L\left(\sum\limits_{n=0}^\infty d_n X_n\right)=-\lambda_m \sum\limits_{n=0}^\infty d_n X_n. $$

For any *k* ≥ 0, we multiply this relation by *wX*_*k*_ and then integrate on [0,1] to obtain

$$ d_k \lambda_k (X_k,wX_k)=d_k \lambda_m (X_k,wX_k). $$

Because (*X*_*k*_,*wX*_*k*_) ≠ 0 and λ_*m *_≠ λ_*k*_ for all *k* ≠ *m*, then *d*_*k*_ = 0, ∀*k *≠ *m*. Hence *X* = *d*_*m*_ *X*_*m*_. This completes the proof. $\square$

### Construction of the solution

In this subsection, we construct the solution and prove its uniqueness. We shall firstly find the general solution of the Fokker–Planck equation (10) by the separation of variables method. Then we shall construct a solution depending on parameters. We shall use (11, 12) to determine the parameters. Finally, we shall verify the solution.

*Step 1* Assume that *u*_0_(*x*,*t*) = *X*(*x*)*T*(*t*) is a solution of the Fokker Planck equation (10). Then we have

$$ \frac{T_t}{T}=\frac{LX}{X}=-\lambda $$

which implies that λ is a constant which is independent of *t*, *x*. From Lemma 3.5, we obtain the general solution of the equation (10) as

$$ u_0(x,t)=\sum\limits_{m\ge 0}c_m X_m(x){\hbox{e}}^{-\lambda_m t}. $$

#### *Remark 3.6*

*u*_0_ is the same as Kimura’s solution (see for example Kimura 1955a,b).

*Step 2* The general solution $u\in H$ of (10) then is

17 $$ u(x,t)=\sum\limits_{m\ge 0}c_m (X_m(x)+a_{m,0}\delta_0(x)+a_{m,1}\delta_{1}(x)){\hbox{e}}^{-\lambda_m t} + b_0 \delta_0(x)+b_1\delta_1(x) $$

where δ_0_ and δ_1_ are the Dirac delta functionals at 0 and 1.

*Step 3* Checking condition (12) with ϕ = 1, ϕ = *x*, ϕ = *wX*_*n*_ yields

$$ \begin{aligned} (u_t,1)&=(u,L^*(1))=0,\\ (u_t,x)&=(u,L^*(x))=0,\\ (u_t,wX_n)&=(u,L^*(wX_n))=-\lambda_n (u,wX_n). \end{aligned} $$

With condition (11), we then obtain

$$ \begin{aligned}1&=(u(\cdot,0),1)=(u(\cdot,\infty),1)=b_0+b_1,\\ p&=(u(\cdot,0),x)=(u(\cdot,\infty),x)=b_1,\\ 1&=(u(\cdot,0),1)=(u,1)=\sum\limits_{m\ge 0} c_m \left(\int\limits_{[0,1]}X_m(x)\,{\hbox{d}}x+a_{m,0}+a_{m,1}\right){\hbox{e}}^{-\lambda_m t} +b_0+b_1,\\ p&=(u(\cdot,0),x)=(u,x)=\sum\limits_{m\ge 0} c_m \left( \int\limits_{[0,1]}xX_m(x)\,{\hbox{d}}x+a_{m,1}\right){\hbox{e}}^{-\lambda_m t} +b_1,\end{aligned} $$

and

18 $$ \begin{aligned} &(u,wX_n)=(u(\cdot,0),wX_n){\hbox{e}}^{-\lambda_n t}=w(p)X_n(p){\hbox{e}}^{-\lambda_n t}.\\ &\Longleftrightarrow c_n(X_n,w X_n){\hbox{e}}^{-\lambda_n t}=w(p)X_n(p){\hbox{e}}^{-\lambda_n t}. \end{aligned} $$

Therefore we have all parameters

19 $$ \begin{aligned} b_1&=p; b_0=1-p\\ a_{m,1}&=-\int\limits_{[0,1]}xX_m(x)\,{\hbox{d}}x; a_{m,0}=-\int\limits_{[0,1]}(1-x)X_m(x)\,{\hbox{d}}x\\ c_n&=\frac{w(p)X_n(p)}{(X_n,wX_n)}. \end{aligned} $$

It follows that the solution should be

20 $$ u(x,t)=\sum\limits_{m \ge 0} c_m X_m(x){\hbox{e}}^{-\lambda_m t}+\left\{1-p+\sum\limits_{m\ge 0}c_m a_{m, 0}{\hbox{e}}^{-\lambda_m t}\right\}\delta_{0}(x) +\left\{p+\sum\limits_{m\ge 0}c_m a_{m,1}{\hbox{e}}^{-\lambda_m t}\right\}\delta_{1}(x) $$

where *X*_*m*_(*x*) is a Gegenbauer polynomial,

21 $$ \begin{aligned} \lambda_m&=\frac{(m+1)(m+2)}{2},\\ a_{m,0}&=-\int\limits_{\Upomega_1}(1-x)X_{m}(x)\,{\hbox{d}}x=-\frac{1}{2},\\ a_{m,1}&=-\int\limits_{\Upomega_1}xX_{m}(x)\,{\hbox{d}}x=(-1)^{m+1}\frac{1}{2},\\ c_m&=\frac{w(p)X_m(p)}{(X_m,wX_m)}=\frac{8w(p)X_m(p)(m+3/2)}{(m+1)(m+2)}. \end{aligned} $$

*Step 4* We will prove the constructed solution *u* satisfies conditions (10, 11, 12). In fact, because in (0, 1),  *u* = *u*_0_, it is clear that *u* satisfies the Fokker Planck equation (10). Moreover, from the representation (20), we have

22 $$ \begin{aligned} (u,1)&=\sum\limits_{m=0}^\infty c_m \left(\int\limits_{[0,1]}X_m(x)\,{\hbox{d}}x+a_{m,0}+a_{m,1}\right){\hbox{e}}^{-\lambda_m t} +1-p+p=1,\\ (u,x)&=\sum\limits_{m=0}^\infty c_m \left(\int\limits_{[0,1]}x X_m(x)\,{\hbox{d}}x+a_{m,1}\right){\hbox{e}}^{-\lambda_m t}+p=p,\\ (u,wX_n)&=c_n (X_n,wX_n){\hbox{e}}^{-\lambda_n t}=w(p)X_n(p){\hbox{e}}^{-\lambda_n t}. \end{aligned} $$

Thus,

23 $$ \begin{aligned} (u(\cdot,0),1)&=1=(\delta_p,1)\\ (u(\cdot,0),x)&=p=(\delta_p,x)\\ (u(\cdot,0),wX_n)&=w(p)X_n(p)=(\delta_p,wX_n). \end{aligned} $$

Because {1, *x*, {*wX*_*n*_}_*n* ≥ 0_} is also a basis of *H*_0_, it follows that

$$ (u(\cdot,0),\phi)=(\delta_p,\phi),\quad \forall \phi \in H_0,$$

i.e. $u(\cdot,0)=\delta_p \in H,$ i.e. *u* satisfies the condition (11).

Finally, from (22) we have

24 $$ \begin{aligned} (u_t,1)&=0=(u,L^*(1))\\ (u_t,x)&=0=(u,L^*(x))\\ (u_t,wX_n)&=w(p)X_n(p)(-\lambda_n){\hbox{e}}^{-\lambda_n t}=-\lambda_n (u,wX_n)=(u,L^*(wX_n)). \end{aligned} $$

Because *L* ^*^ is linear and {1, *x*, {*wX*_*n*_}_*n* ≥ 0_} is also a basis of *H*_0_, it follows that

$$ (u_t,\phi)=(u,L^*(\phi)),\quad \forall \phi\in H_0, $$

i.e. *u* satisfies the condition (12).

Therefore, *u* is a solution of the Fokker–Planck equation associated with the Wright–Fisher model, indeed.

We can easily see that this solution is unique. In fact, assume that *u*_1_,*u*_2_ are two solutions of the Fokker–Planck equation associated with Wright–Fisher model. Then *u* = *u*_1_ − *u*_2_ satisfies

$$ \begin{aligned} u_t&=Lu \hbox { in }(0,1)\times (0,\infty),\\ u(x,0)&=0 \hbox { in }(0,1);\\ (u_t,\phi)&=(u,L^* \phi), \forall \phi\in H_0. \end{aligned} $$

Therefore

$$ \begin{aligned} (u_t,1)&=(u,L^*(1))=0,\\ (u_t,x)&=(u,L^*(x))=0,\\ (u_t,wX_n)&=(u,L^*(wX_n))=-\lambda_n (u,wX_n). \end{aligned} $$

Therefore

$$ \begin{aligned} (u,1)&=(u(\cdot, 0),1)=0,\\ (u,x)&=(u(\cdot,0),x)=0,\\ (u,wX_n)&=(u(\cdot,0),wX_n){\hbox{e}}^{-\lambda_n t}=0. \end{aligned} $$

Because {1, *x*, {*wX*_*n*_}_*n* ≥ 0_} is also a basis of *H*_0_, it follows that $u=0 \in H.$

Altogether, we obtain our main result.

#### **Theorem 3.7**.

*The Fokker–Planck equation associated with Wright–Fisher model possesses a unique solution*.

**Fig. 1:**
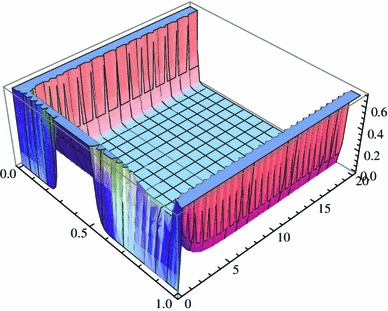
Behaviour of the new solution from δ_*p*_ to *p*δ_1_ + (1 − *p*)δ_0_ in time with *p* = 0.4

This new solution continuously deforms the initial state δ_*p*_(*x*) (the allele *A*_1_ has relative frequency *p*) to *p*δ_1_(*x*) + (1 − *p*)δ_0_(*x*) (allele *A*_1_ is fixed with probability *p* and *A*_2_ is fixed with probability 1 − *p*) as time proceeds from 0 to $\infty.$ In fact, the sequence {*u*_*m*_(*x*,*t*)}_*m* ≥ 0_ satisfying

25 $$ \begin{aligned} u_m(x,t)&=\sum\limits_{i=0}^m c_i X_i(x){\hbox{e}}^{-\lambda_i t}\\ &\quad +\left\{1-p+\sum\limits_{i=0}^m c_i \left(-\frac{1}{2}\right){\hbox{e}}^{-\lambda_i t}\right\} \frac{m}{\sqrt{2\pi}}{\hbox{e}}^{-x^2 m^2/2}\\ &\quad +\left\{p+\sum\limits_{i= 0}^m c_i \left(\frac{(-1)^{i+1}}{2}\right){\hbox{e}}^{-\lambda_i t}\right\} \frac{m}{\sqrt{2 \pi}}{\hbox{e}}^{-(1-x)^2 m^2/2} \end{aligned} $$

tends to *u* for $m\to \infty.$ Therefore, we can visualise the asymptotic behaviour with the help of Mathematica (Fig. 1).

**Fig. 2:**
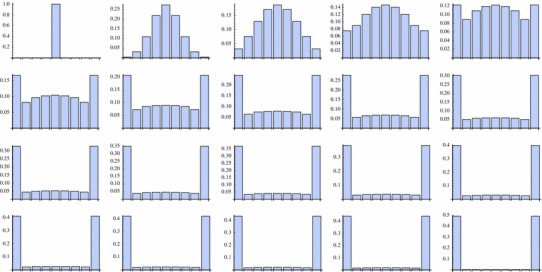
Behaviour of the discrete solution in time $k=0,1,\ldots,18$ and *k* = 32 with *p* = 0.5

**Fig. 3:**
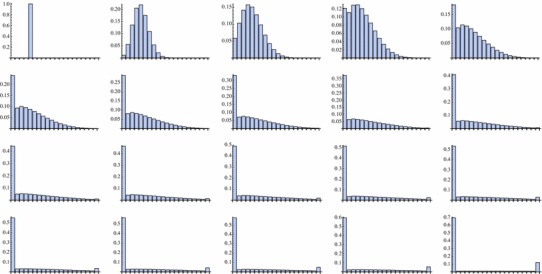
Behaviour of the discrete solution in time $k=0,1,\ldots,18$ and *k* = 30 with *p* = 0.25

This behaviour coincides with the discrete one (Figs. 2, 3): 

## Applications

Our global solution readily yields the quantities of interest of the evolution of the process (*X*_*t*_)_*t* ≥ 0_ such as the expectation and the second moment of the absorption time, *m*th moments, fixation probabilities, the probability of coexistence, or the probability of heterogeneity.

### Absorption time

Let *V*_0_ : = {0,1} be the domain representing a population of 1 allele. Here, 0 corresponds to the loss of *A*_1_, that is, the fixation of *A*_2_, and 1 corresponds to the opposite situation. Either of these irreverible events is called an absorption.

We denote by $T^1_2(p)=\inf \{t>0: X_t\in V_0|X_0=p \}$ the first time when the population has only 1 allele left, that is, when absorption occurs. *T*_2_^1^(*p*) is a continuous random variable valued in $[0,\infty)$ with probability density function denoted by ϕ(*t*, *p*). *V*_0_ is invariant (absorption set) under the process *X*_*t*_, i.e. if $X_s \in V_0$ then $X_t\in V_0$ for all *t* ≥ *s*. We have

$$ {\mathbb{P}}(T^1_2(p)\le t)={\mathbb{P}}(X_{t}\in V_0|X_0=p) $$

It follows that

$$ \phi(t,p)=\int\limits_{V_0}\frac{\partial}{\partial t} u(x,p,t)\,{\hbox{d}}x. $$

Therefore the expectation of the absorption time for having only one allele is

$$ \begin{aligned} {\mathbb{E}}(T^1_2(p))&=\int\limits_0^\infty t\phi(t,p)\,{\hbox{d}}t\\ &= \int\limits_{V_0}\left(\int\limits_0^\infty t\frac{\partial}{\partial t} u(x,t)\,{\hbox{d}}t \right)\,{\hbox{d}}x\\ &=\sum\limits_{m=0}^{\infty}\sum\limits_{V_0}(-\lambda_m)c_m (X_m(x)+a_{m,0}\delta_{e_0}(x)+a_{m,1}\delta_{e_1}(x))\left( \int\limits_0^\infty t{\hbox{e}}^{-\lambda_m t}\,{\hbox{d}}t\right)\,{\hbox{d}}x\\ &=-\sum\limits_{m=0}^\infty \frac{1}{\lambda_m} c_{m} (a_{m,0}+a_{m,1} )\\ &=\sum\limits_{m=0}^\infty \frac{1}{\lambda_{2m}} c_{2m}\\ &=\sum\limits_{m=0}^\infty 16 p(1-p)(2 m +3/2)/{(2 m+1)^2(2 m+2)^2} X_{2m}(p). \end{aligned} $$

and its second moment is

26 $$ \begin{aligned} {\mathbb{E}}(T^1_2(p))^2&=\int\limits_0^\infty t^2\phi(t,p)\,{\hbox{d}}t\\ &= \int\limits_{V_0}\int\limits_0^\infty t^2\frac{\partial}{\partial t} u(x,p,t)\,{\hbox{d}}t\,{\hbox{d}}x\\ &= \sum\limits_{m=0}^{\infty} \int\limits_{V_0} (-\lambda_m)c_m ( X_m(x)+a_{m,0}\delta_{e_0}(x)+a_{m,1}\delta_{e_1}(x)) \int\limits_0^\infty t^2{\hbox{e}}^{-\lambda_m t}\,{\hbox{d}}t\,{\hbox{d}}x\\ &=-\sum\limits_{m=0}^\infty \frac{2}{\lambda^2_m} c_{m} ( a_{m,0}+a_{m,1} )\\ &=\sum\limits_{m=0}^\infty \frac{2}{\lambda^2_{2m}}c_{2m}\\ &=\sum\limits_{m=0}^\infty 64 p(1-p)(2 m +3/2)/{(2 m+1)^3(2 m+2)^3} X_{2m}(p). \end{aligned} $$

#### *Remark 4.1*

${\mathbb{E}}(T^1_2(p))=-2\{p \ln(p) +(1-p) \ln(1 - p)\}$ is the unique solution of the one-dimensional boundary value problem

$$ \left\{\begin{array}{l} Lv=-1, \hbox { in (0,1)}\\ v(0)=v(1)=0. \end{array}\right. $$

**Fig. 4:**
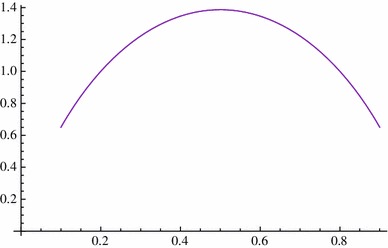
Comparison results of expectation of the absorption time

We easily check that this agrees with our formula above by using Mathematica (Fig. 4):

### *n*th moments

By induction, it is easy to prove that

$$ \int\limits_{[0,1]}x^n X_{m-1}(x)\,{\hbox{d}}x=(-1)^{m}\frac{1}{2}\left\{ \frac{(n-1)\ldots(n-m)}{(n+1)\ldots(n+m)}-1\right\}. $$

Therefore, the *n*th moment is

$$ \begin{aligned} m_n(t)&=(u,x^n)\\&=\sum\limits_{m=0}^\infty c_m \left(\int\limits_{[0,1]}x^n X_m(x)\,{\hbox{d}}x \right){\hbox{e}}^{-\lambda_m t} +\left(p+\sum\limits_{m=0}^\infty c_m a_{m,1}{\hbox{e}}^{-\lambda_m t}\right)\\&=p+\sum\limits_{i=1}^\infty c_{i-1}\left( \int\limits_{[0,1]}x^n X_{i-1}(x)\,{\hbox{d}}x+a_{i-1,1}\right){\hbox{e}}^{-\lambda_{i-1} t}\\ &=p+\sum\limits_{i=1}^\infty \frac{2(2i+1)}{i(i+1)} p(1-p) (-1)^i X_{i-1}(p) \frac{(n-1)\ldots(n-i)}{(n+1)\ldots(n+i)}{\hbox{e}}^{-\frac{i(i+1)}{2}t}. \end{aligned} $$

This *n*th moment coincides with Kimura’s (1955) one.

### Fixation probabilities and probability of coexistence of 2 alleles

The fixation probability for *A*_2_ (loss of *A*_1_) is

$$ \begin{aligned} {\mathbb{P}}(X_t=0|X_0=p)&=\int\limits_{\{0\}}u(x,t)\,{\hbox{d}}x\\ &=1-p+\sum\limits_{m=0}^\infty c_m a_{m,0}{\hbox{e}}^{-\lambda_m t}\\ &=1-p-\frac{1}{2}\sum\limits_{m=0}^\infty \frac{8w(p)X_m(p)(m+3/2)}{(m+1)(m+2)}{\hbox{e}}^{-\lambda_m t}. \end{aligned} $$

Analogously, the fixation probability of *A*_1_ is

$$ \begin{aligned} {\mathbb{P}}(X_t=1|X_0=p)&=\int\limits_{\{1\}}u(x,t)\,{\hbox{d}}x\\&=p+\sum\limits_{m=0}^\infty c_m a_{m,1}{\hbox{e}}^{-\lambda_m t}\\&=p-\frac{1}{2}\sum\limits_{m=0}^\infty (-1)^m\frac{8w(p)X_m(p)(m+3/2)}{(m+1)(m+2)}{\hbox{e}}^{-\lambda_m t}. \end{aligned} $$

The probability of coexistence of the 2 alleles *A*_1_, *A*_2_ therefore is

$$ \begin{aligned} {\mathbb{P}}(X_t\in (0,1)|X_0=p)&=\int\limits_{(0,1)}u(x,t)\,{\hbox{d}}x\\ &=\sum\limits_{m=0}^\infty c_m \int\limits_{(0,1)} X_m(x)\,{\hbox{d}}x{\hbox{e}}^{-\lambda_m t}\\ &=\sum\limits_{m=0}^\infty c_{2m}{\hbox{e}}^{-\lambda_{2m} t}\\ &=\sum\limits_{m=0}^\infty \frac{8w(p)X_{2m}(p)(2m+3/2)}{(2m+1)(2m+2)}{\hbox{e}}^{-\lambda_{2m} t}. \end{aligned} $$

These three probabilities sum to 1, as they should.

**Fig. 5:**
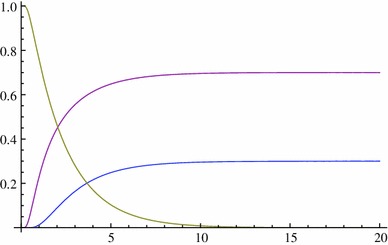
*p* = 0.3

**Fig. 6:**
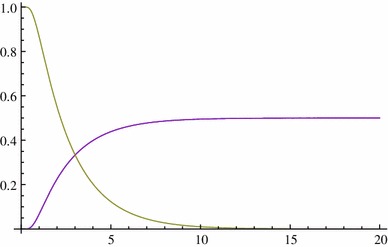
*p* = 0.5

We consider their behaviour for *p* = 0.3 and *p* = 0.5 (Figs. 5, 6):

#### *Remark 4.2*

(i) $${\mathbb{P}(X_t\in [0,1]|X_0=p)=\mathbb{P}(X_t=0|X_0=p)+\mathbb{P}(X_t=1|X_0=p)+ \mathbb{P}(X_t\in (0,1)|X_0=p)=1;}$$
(ii) ${\mathbb{P}(X_t=0|X_0=p)}$ and ${\mathbb{P}(X_t=1|X_0=p)}$ increase quickly in $t\in(0,5)$(10N generations) from 0 and then tend slowly to 1 − *p* and *p*, respectively;
(iii) When *p* = 0.5, the situation is symmetric between the two alleles, that is, ${\mathbb{P}(X_t=0|X_0=0.5)=\mathbb{P}(X_t=1|X_0=0.5). }$

### Heterogeneity

The probability of heterogeneity is

$$ \begin{aligned} H_t&=\int\limits_{[0,1]} 2x(1-x)u(x,t)\,{\hbox{d}}x\\ &=2(u,wX_0)\\ &=2(c_0X_0,wX_0){\hbox{e}}^{-\lambda_0 t}\\ &=2w(p)X_0(p){\hbox{e}}^{-t}\\ &=H_0{\hbox{e}}^{-t}. \end{aligned} $$

Of course, this goes to 0 for $t\to \infty,$ as it should.

## Conclusion

We have constructed a unique global solution of the Fokker–Planck equation associated with the Wright–Fisher model. This solution leads to explicit formulae for the absorption time, fixation probabilities, the probability of coexistence, *n*th moments, heterogeneity, and other quantities.

## Footnotes

1 Here is a remark for readers not familiar with this mathematical construction: This is a formal definition, as *δ*_*p*_ defined in this manner is not a function itself, but rather operates on continuous functions by assigning to them their value at the particular point *p*. Thus, while the product (*f*, *g*) had been first defined for square integrable functions *f*, *g*, we now apply it to the pair (*δ*_*p*_, *ϕ*) where *δ*_*p*_ is a more general object and in turn *ϕ* is a more restricted function (continuous instead of simply square integrable).

2 The Gegenbauer polynomials generalise other important classes of polynomials, like the Legendre and the Chebyshev polynomials, and they constitute in turn special cases of the Jacobi polynomials.
